# Measuring the psychosocial health of adolescent and young adult (AYA) cancer survivors: a critical review

**DOI:** 10.1186/1477-7525-8-25

**Published:** 2010-03-06

**Authors:** Tara Clinton-McHarg, Mariko Carey, Rob Sanson-Fisher, Anthony Shakeshaft, Kathy Rainbird

**Affiliations:** 1 Health Behaviour Research Group, Priority Research Centre for Health Behaviour (PRCHB), University of Newcastle, Callaghan, New South Wales, Australia; 2 National Drug and Alcohol Research Centre (NDARC), University of New South Wales, Sydney, New South Wales, Australia; 3 Health Research Consultant, Baldivis, Western Australia, Australia

## Abstract

**Background:**

Adolescent and young adult (AYA) cancer survivors require psychometrically rigorous measures to assess their psychosocial well-being. Without methodologically adequate scales the accuracy of information obtained on the prevalence of needs, predictors of risk, and the potential success of any interventions, can be questioned. This review assessed the psychometric properties of measures designed specifically to identify the psychosocial health of this unique population.

**Methods:**

Medline, PsycINFO, CINAHL and EMBASE databases were searched to identify measures developed to assess the psychosocial health of AYA cancer survivors. Searches were limited to the years 1998-2008. A search of Medline revealed that the number of publications related to the assessment of psychosocial well-being in AYA cancer survivors prior to this period were minimal. The psychometric properties of identified measures were evaluated against pre-determined and generally accepted psychometric criteria including: reliability (internal consistency and test-retest); validity (face, content, construct, and criterion); responsiveness; acceptability; and feasibility.

**Results:**

Seven quality of life measures met the inclusion criteria. No measures of unmet need were identified. All seven measures reported adequate internal consistency, face, content, and construct validity. Test-retest reliability, criterion (predictive) validity, responsiveness, acceptability, and feasibility were rarely examined.

**Conclusions:**

There is a need to further evaluate the psychometric properties of existing quality of life measures for AYA cancer survivors. Valid, reliable, and acceptable measures which can assess the psychosocial needs of this population should also be developed.

## Background

### The global burden of adolescent and young adult cancer

Cancer is the leading disease-related cause of mortality among adolescents and young adults (AYAs) resulting in approximately 134,000 deaths worldwide, each year [1]. AYAs have been broadly defined as young people between the ages of 15 and 30 years [2-4]. Advances in treatment mean that between 73-82% of AYA diagnosed with cancer will now survive up to five years post-diagnosis [5-8]. Increasing survival rates mean that a greater number of AYAs are living longer with the psychosocial sequelae of their cancer diagnosis and its treatment [7-10]. AYAs not only experience the wide range of physical, psychological, social and spiritual concerns of cancer survivors of all ages, but often have additional and unique needs due to their cancer occurring during a crucial stage of their personal and social development [11-13].

### Diversity of AYAs with cancer

Cancer survivorship has been defined as beginning from the time of cancer diagnosis and includes people at various stages of the disease trajectory [14]. Although grouped due to their unique developmental phase, AYA cancer survivors represent a variety of socio-demographic backgrounds and cancer types. Some AYA survivors include students who live with their families, while others are employed and live independently [15,16]. The majority have a history of lymphoma, leukaemia, invasive skin, genital, endocrine, brain or bone cancer [4,6-8].

### The acute psychosocial impact of cancer and its treatment

The acute psychosocial impact of cancer and its treatment may be substantial. Some AYAs experience physical side-effects such as pain, vomiting, and nausea [17,18]. These physical symptoms can lead to high levels of distress in young people, and can limit their ability to engage in normal activities such as attending school or work [15]. Participation in social events is often restricted, and can mean that normal adolescent rites of passage, such as the formation of identity and independence are inadequately achieved [12,19,20]. This lack of social interaction with peers can lead to feelings of isolation and loneliness [11,12]. Side-effects of treatment such as weight loss, hair loss or impaired physical development can impact on perceived body image and can contribute to loss of self-confidence [12,19,21]. Feelings of hopelessness or anxiety have also been reported [2,22]. A young person's cancer diagnosis can also lead to changes in family dynamics and impact on their relationships with parents, siblings, and significant others [12,13,19].

### The long-term psychosocial impact of cancer and its treatment

Although some acute psychosocial consequences cease once treatment is completed, others can have a long-term impact on the psychosocial health of the survivor. Compared with other young people their age, some long-term AYA cancer survivors report poorer health outcomes including higher rates of obesity, anxiety and depression [20,23,24]. Some also experience cognitive impairment which can impact on employment and educational attainment [25,26]. Concerns related to reduced fertility and sexual dysfunction are also prevalent among AYA cancer survivors [27,28].

### Approaches to assessing the psychosocial health of cancer survivors

The widely accepted World Health Organisation (WHO) definition of health encompasses physical, mental and social aspects of well-being, all of which are inextricably linked and contribute to the global health of the individual [6]. This necessitates the use of multi-dimensional rather than uni-dimensional measures in order to develop a comprehensive assessment of the health of an individual [29]. Multi-dimensional measures of health assess elements of physical, psychological, social, and often spiritual well-being [29]. For cancer patients these generally include measures of quality of life (QoL) and perceived need. QoL measures assess an individual's perception of their current health status compared with their health expectations [29,30]. In contrast, measures of perceived need identify the needs individuals regard as being unmet and the magnitude of help likely to be required to address them [31,32]. While there are a number of QoL and unmet needs tools for adult cancer patients and survivors [33,34], few measures specific to AYA cancer survivors have been identified [35-39]. Given the unique needs and experiences of this group, psychosocial health measures developed and validated with this population are needed to accurately assess well-being.

Self-report rather than proxy measurement is generally preferred for assessing psychosocial health. Although proxy measurement may allow for the inclusion of patients who are too ill or do not have the necessary literacy skills to participate alone, proxies can tend to base their assessment on their impression of the patient, rather than the actual situation [40,41]. Proxies are also more inclined to focus on negative or extreme behaviour rather than positive or usual behaviour [41].

As well as being assessed by self-report and covering broad psychosocial domains, measures designed to assess the psychosocial well-being of AYA cancer survivors need to be able to accurately reflect the unique experiences of this population. Such measures should be able to capture, and be sensitive to, changes in psychosocial health across the disease trajectory so that the effectiveness of interventions can be assessed [38]. Measures also need to be psychometrically robust so that the prevalence of needs, and subgroups of young people experiencing high needs, can be accurately identified [38].

The aim of this review is to critically examine the psychometric properties of multi-dimensional, self report measures developed to assess the psychosocial health of AYA cancer survivors.

## Methods

### Database search to identify relevant publications

Medline, PsychINFO, EMBASE and CINAHL databases were searched to identify publications which described the development of measures for assessing psychosocial outcomes in AYA cancer survivors. These databases were chosen as they all provide extensive coverage of journals in the field of cancer research.

**Figure 1:**
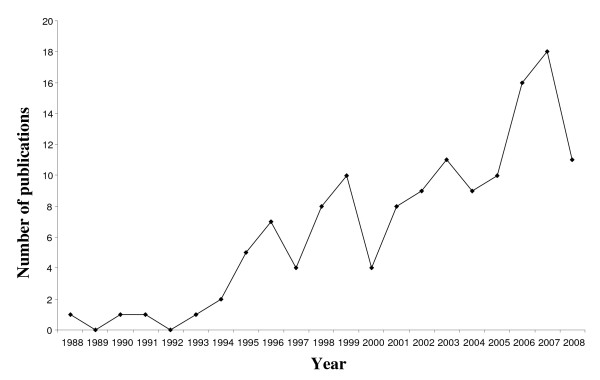
**Number of publications related to the assessment of psychosocial well-being in AYA cancer survivors by year (1988-2008)**.

The database search was performed using the following combinations of keywords: [neoplasm or cancer or oncol*] and [adoles* or teenager or young adult or youth] and [perceived need* or unmet need* or quality of life or psychosocial or distress] and [develop* or questionnaire or survey or measure or scale] and [psychometric or reliability or validity or acceptability]. Results of the search were limited to the English language and covered the last ten years from 1998 to 2008. This timeframe was selected as a preliminary search of Medline for all AYA related psychosocial research without a year limitation revealed that there had been minimal (< 17%) research output in field prior to 1998 (Figure 1), with only one publication before 1988 identified (one publication in 1976). Appraisal of these 23 publications revealed that no additional measures met the inclusion criteria (outlined below) prior to 1998.

Duplicate publications, and publications which did not specifically describe the development, psychometric properties, or acceptability of a measure, were excluded. Full text articles of the remaining publications were obtained and reviewed to identify relevant measures.

### Inclusion and exclusion of measures

While AYAs are commonly defined as 15-30 year olds, definitions in the literature vary [2-4]. Therefore, an inclusive approach was employed whereby scales developed for use with young people less than 15 years but with an upper age limit between 15 and 30 years were included (eg 12-20 years). Similarly, scales developed for use with populations older than 15 years but less than 30 years were included (eg 16-28 years).

Measures which met all of the following criteria were included in the study for coding: 1) quantitative; 2) developed or validated in English; 3) multi-dimensional and measured at least the following three psychosocial domains: physical, psychological, and social; 4) cancer specific; 5) assessed the well-being of patients or survivors; 6) developed specifically for AYA or included participants aged between15-30 years in their sample; and 7) completed by self-report.

After identifying measures which met all of the inclusion criteria, a second search of all databases by 'measure name' was performed to ensure that all publications relating to each identified measure were obtained.

### Measure coding

#### Sample characteristics

In order to accurately assess the psychometric properties of a measure, the sample used to develop the measure should be described [42]. Measure development papers were examined to determine whether the following sample characteristics were reported: a) inclusion and exclusion criteria; b) setting; c) response rate; d) sample size; e) age of participants; f) proportion of male and female participants; g) cancer type; and h) cancer treatment stage.

#### Psychometric properties

Measures were coded using pre-defined criteria considered important for scale development and health outcome measurement [42-51]. The rigorousness of each measure was assessed against criteria for: a) reliability; b) validity; c) responsiveness; d) acceptability; e) feasibility; and f) cross-cultural adaptation, summarised in Table 1.

**Table 1:** Summary of psychometric properties and criteria used to review measures.

Psychometric Property	Criteria
***Reliability***	
*Internal consistency* degree to which responses to all items on a scale are consistent [43]	Calculated correlations for total scale and domains [44] - Cronbach's alpha (*α*) > 0.70 [42,44] - Kuder-Richardson 20 (KR-20) > 0.70 [42,44]
*Test-retest* reproducibility of scores on a scale over repeated administrations [44]	Second administration within 2-14 days [46] Calculated correlations for total scale, domains and items [47] - Cohen's kappa coefficient (*κ*) > 0.60 [44] - Pearson correlation coefficient (*r*) > 0.70 [42,44] - Intraclass correlation coefficient (ICC) > 0.70 [42,44]
***Validity***	
*Face* subjective assessment of whether a scale 'appears' to measure what it is designed to measure [43]	Assessed as reasonable by those who administer/complete it [43]
*Content* degree to which the content of a scale is representative of the issue being measured [43]	Reported item selection process [42,44] Content assessed by experts [42,44] Reported which aspects of the measure were revised [42,44]
*Construct* way in which the internal structure of a scale relates to other conceptual constructs [44]	Stated hypothesis about correlations between measures [44] - Convergent (*r*) > 0.40 or Divergent (*r*) < 0.30 [48] Calculated correlations between known-groups [42] Performed factor analysis [44] - Eigenvalues > 1 [49]
*Criterion* how well a scale agrees with existing "gold standard" measurement of the same issue [44]	Provided rationale for "gold standard" measure [44] Stated type of criterion validity (concurrent or predictive) [43] Reported proportions [44,50] - Sensitivity - % with issue correctly classified [44,50] - Specificity - % without issue correctly classified [44,50]
***Responsiveness*** sensitivity of a scale to detect clinically important change in an outcome or behaviour over time [42,50]	Reported floor/ceiling effects [51] - < 5% of respondents have highest or lowest score [51] Reported magnitude of change [42] - Effect size > 0.5 [42,44,50]
***Acceptability*** level of burden placed on those who complete the measure [42]	Reported response rate, missing items, reading level, time to complete [42]
***Feasibility*** level of burden placed on those who administer the measure [42]	Reported perceived time to administer, score, interpret [42]
***Cross-cultural adaptation *** conceptually, linguistically equivalent and display similar psychometric properties to the original form [42]	Confirmed reliability and validity reflects the original version [42]

### Inter-rater agreement of coding existing measures

One reviewer used the inclusion and exclusion criteria to identify measures for inclusion in the review. A second reviewer cross-checked 15% of the measures, to confirm their inclusion and exclusion status. The psychometric criteria of all included measures were reviewed by the first author and checked by the second.

## Results

### Database search to identify relevant publications

The initial search of the Medline, PsychINFO, EMBASE and CINAHL databases identified a total of 552 publications related to assessing psychosocial outcomes in AYA cancer survivors, with 436 papers having been published in the last ten years (1998-2008). Of these 436 publications, 91 were duplicates and 146 did not describe the development of a measure. The remaining 199 publications described the development of 204 measures.

**Figure 2:**
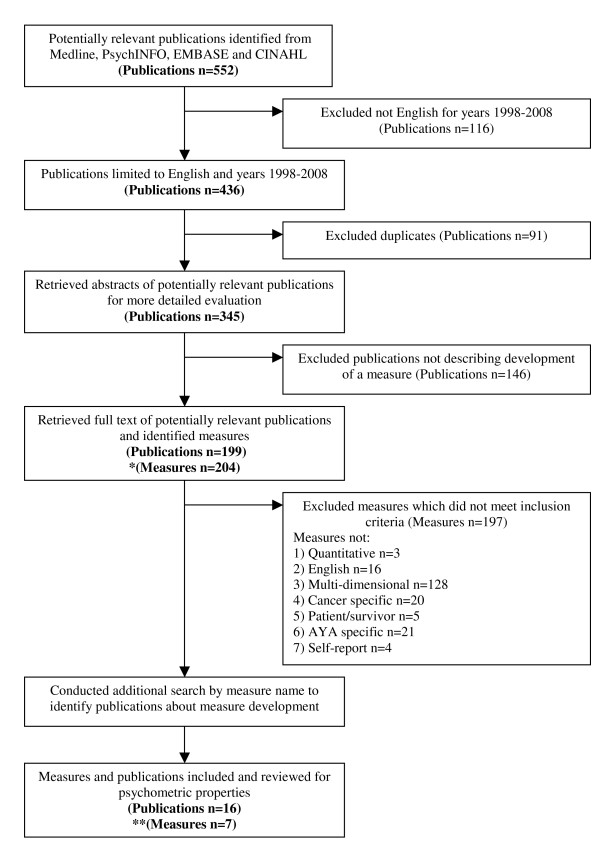
**Flowchart of the publication and measure inclusion and exclusion process**. *Some publications described the development ofmore than one measure. ** Development of some measures were reported across more than one publication.

197 measures did not meet the inclusion criteria (Figure 2), leaving seven measures to be included in the psychometric review. These included the: 1) Adolescent Quality of Life Instrument (AQoL)[35,36]; 2) Minneapolis-Manchester Quality of Life Instrument (MMQL) - Adolescent Form [52-54]; 3) Pediatric Quality of Life Inventory (PedsQL) 3.0 Cancer Module Child and Adolescent (C&A) Forms [55-58]; 4) Quality of Life - Cancer Survivors (QOL-CS) validation in childhood cancer survivors [16]; 5) Pediatric Cancer Quality of Life Inventory - 32 Short Form (PCQL-32) [59-61]; 6) Pediatric Cancer Quality of Life Inventory (PCQL) Modular Approach [62]; and 7) Perceived Illness Experience Scale (PIE)[63,64].

Six measures were developed in the United States, one was developed in the United Kingdom [63,64]. A description of each measure's domains and number of items is presented in Table 2.

**Table 2:** Items and domains of measures included in the review.

Measure	Items	Domains	Description	Reference
**AQoL** Adolescent Quality of Life Instrument	16	5	normal activities, social/family interactions, health status, mood, meaning of being ill	[35,36]
**MMQL Adolescent Form** Minneapolis-Manchester Quality of Life Instrument	46	7	physical, psychological, social, and cognitive functioning, body image, outlook on life, intimate relations	[52-54]
**PedsQL 3.0 Cancer Module (C&A) **Pediatric Quality of Life Inventory Child and Adolescent Forms	27	8	pain and hurt, nausea, procedural anxiety, treatment anxiety, worry, cognitive problems, perceived physical appearance, communication	[55-58]
**QOL-CS** Quality of Life-Cancer Survivors	41	4	physical, psychological (distress and fear), social, and spiritual well-being	[16]
**PCQL-32** Pediatric Cancer Quality of Life Inventory - 32 Short Form	32	5	disease and treatment-related symptoms, physical, psychological, social, and cognitive functioning	[59-61]
**PCQL Modular Approach **Pediatric Cancer Quality of Life Inventory Modular Approach	23	5	(core) physical, psychological, social, (modules) pain, nausea	[62]
**PIE** Perceived Illness Experience Scale	34	9	physical appearance, interference with activity, peer rejection, integration in school, manipulation, parental behaviour, disclosure, preoccupation with illness, impact of treatment	[63,64]

### Sample characteristics

Overall, reporting of the sample accrual method and the sociodemographic and clinical characteristics of participants for each measure was comprehensive (Table 3). Of the seven measures, three did not report a response rate, one did not describe the inclusion and exclusion criteria, and one measure did not report the proportion of male and female participants or cancer type.

**Table 3:** Reported sample characteristics for each measure.

	Sample characteristics							
	
Measure	Inclusion/exclusion	Setting	Response rate (%)	Sample size (n)	Age (yrs)	Gender (%)	Cancer type (%)	Cancer treatment stage (%)
**AQoL **[35]	Reported	Hematology /oncology clinic	95	75	9-20 mean 12.4	M (55) F (45)	Leukaemia (50) Bone/joint (17) Lymphomas (9) Neurological (9) Hodgkin's (5) Other (9)	In treatment (55) Pre or post treatment (45)
**MMQL Adolescent Forrm **[52]	-	Nine hospitals	-	268	13-20.9 median 16.6	M (56) F (44)	Leukaemia ALL (37) Leukaemia AML (8) Hodgkin's (11) Non-Hodgkin's (11) Brain (6) Other (27)	On therapy (41) Off therapy > 1 year (59)
**PedsQL 3.0 Cancer Module (C&A) **[55]	Reported	Hematology/oncology center and Center for Cancer and Blood Diseases	-	220	5-18 mean 10.9	M (56) F (44)	Leukaemia (50) Brain (7) Non-Hodgkin's (6) Hodgkin's (3) Wilm's Tumor (6) Other (28)	On treatment (54) Off treatment < 1 year (18) Off treatment > 1 year (28)
**QOL-CS **[16]	Reported	University medical center	53	176	16-28 mean 21.8	M (43) F (57)	Leukaemia (30) Brain/CNS (11) Lymphoma (21) Wilm's Tumor (10) Sarcomas (16) Other (11)	3-27 yrs post-diagnosis (100) (average 13.3 yrs)
**PCQL-32 **[59]	Reported	Three pediatric cancer centers	89.5	291	8-18 mean 11.78	M (61) F (39)	Leukaemia ALL (44) Leukaemia AML (6) Leukaemia other (1) Hodgkin's (6) Non-Hodgkin's (9) Other (34)	Newly on-treatment (37) Relapsed on treatment (8) Remission off-treatment (11) Long-term off-treatment (44)
**PCQL Modular Approach **[62]	Reported	Three pediatric cancer centers	89.5	291	8-18 mean 11.78	-	-	On treatment (45) Off treatment (55)
**PIE **[63]	Reported	Children's cancer unit	-	41	8-24 mean 14.6	M (49) F (51)	Leukaemia ALL (68) Wilm's Tumor (15) Sarcomas (12) Non-Hodgkin's (5)	Maintenance treatment (41) Follow-up only (59)

*Data taken from the publication referenced in the Measure column unless otherwise referenced within the table.

All measures were developed using samples recruited through hospitals or medical centres. Sample sizes ranged from 41-291 participants, and age of participants ranged from 5-28 years (mean range of 10.9-21.8 years). The proportion of males and females was reasonably equally distributed. For the majority of studies, the greatest proportion of young people had been diagnosed with Leukaemia. Cancer treatment stage ranged from newly on treatment to 3-27 years post-diagnosis.

### Psychometric properties

An overall summary of the psychometric properties reported for each measure can be seen in Table 4.

**Table 4:** Summary of psychometric properties reported for each measure.

Measure	Internal consistency	Test-retest reliability		Face/content validity	Construct validity			Responsiveness	Acceptability	Cross-cultural
							
		Time	ICC		Convergent/divergent	Known groups	Factor analysis			
**AQoL**	√	√	-	√	-	√	√	-	√	-
**MMQL Adolescent Form**	√	√	√	√	√	√	-	-	-	√
**PedsQL 3.0 Cancer Module (C&A)**	√	-	-	√	√	√	-	-	√	√
**QOL-CS**	√	-	-	-	√	√	√	-	√	-
**PCQL-32**	√	-	-	√	√	√	-	√	√	-
**PCQL Modular Approach**	√	-	-	√	-	√	-	√	√	-
**PIE**	√	-	-	√	√	√	-	-	√	-

### Reliability

#### Internal consistency

Table 5 shows five measures had at least one domain with poor internal consistency (Cronbach's alphas < 0.70), although their total scale internal consistency was adequate. Two measures did not report internal consistency for their domains (AQoL and PCQL Modular Approach), however both the pain and nausea modules of the PCQL Modular Approach had a Cronbach's alpha > 0.70.

**Table 5:** Coding of reliability criteria for each measure.

Measure	Internal consistency		Test-retest reliability		
		
	n	Cronbach's alpha α > 0.70	n	Administration Period	Intraclass correlation ICC > 0.70
**AQoL **[35]	75	Total scale = 0.77 No domains reported	17	Pre-weekend to post-weekend Post-weekend to one month [36]	-
**MMQL Adolescent Forrm **[52]	397	Total scale = 0.78 6/7 domains > 0.70 Physical = 0.88 Psychological = 0.83 Social = 0.81 Cognitive = 0.89 Body image = 0.80 Outlook = 0.85	87	Two week interval	Total scale = 0.71 5/7 domains > 0.70 Physical = 0.90 Cognitive = 0.88 Body image = 0.73 Outlook = 0.76 Relations = 0.81
**PedsQL 3.0 Cancer Module (C&A) **[55]	220	Total scale = 0.72 6/8 domains > 0.70 Pain and hurt = 0.70 Nausea = 0.79 Procedural Anxiety = 0.82 Treatment Anxiety = 0.79 Worry = 0.74 Cognitive = 0.76	-	-	-
**QOL-CS **[16]	176	Total Scale = 0.87 5/6 domains > 0.70 Physical = 0.81 Psychological = 0.82 Fears = 0.88 Social = 0.76 Spiritual = 0.78	-	-	-
**PCQL-32 **[60]	291	Total scale = 0.91 4/5 domains > 0.70 Disease/treatment = 0.83 Physical = 0.78 Psychological = 0.76 Cognitive = 0.81	-	-	-
**PCQL Modular Approach **[62]	281	Total scale = 0.83 No domains reported All modules > 0.70 Pain = 0.82 Nausea = 0.71	-	-	-
**PIE **[63]	41	Total scale = 0.84 2/9 domains > 0.70 Manipulation = 0.70 Parental behaviour = 0.73 Total scale = 0.91 4/9 domains > 0.70 Peer rejection = 0.79 Parental behaviour = 0.71 Preoccupation illness = 0.73 Food = 0.70 [64]	41	-	-

*Data taken from the publication referenced in the Measure column unless otherwise referenced within the table.

#### Test-retest

Two measures examined test-retest reliability. For both studies, the second administration of the measure was within the recommended time-frame of 2-14 days. Only the MMQL Adolescent Form reported the intraclass correlations for the two administrations, with five of the seven domains having intraclass correlations > 0.70.

### Validity

#### Face/content

Table 6 shows six of the seven measures explored face and content validity, with most involving both AYA cancer survivors and health care providers in their development.

**Table 6:** Coding of validity criteria for each measure.

Measure	Face/Content validity	Construct validity		
		
		Convergent *r *> 0.40 Divergent *r *< 0.30	Known groups (discriminate)	Factor Analysis Eigenvalues > 1
**AQoL **[35]	Assessed by survivors Review of literature Item wording, redundancy Pilot test (n = 7)	-	Receiving treatment (n = 41) Not receiving treatment (n = 34) P = 0.000	6 factors Represented 66.5% of variance
**MMQL Adolescent Forrm **[52]	Assessed by survivors Focus group (n = 20) Interviews (n = 20) Pilot test 1^st ^(n = 10) 2^nd ^(n = 10)	Child Health Questionnaire - Child Form Hypotheses supported 42 correlations > 0.40	Healthy adolescents (n = 129) On therapy (n = 110) Off therapy (n = 158) P < 0.05 for 4 domains	-
**PedsQL 3.0 Cancer Module (C&A) **[55]	Adapted from Pediatric Cancer Quality of Life Inventory (PCQL), PedsQL 1.0 Cancer Module, and PedsQL	PedsQL 4.0 Generic Core Scale PedsQL Multidimensional Fatigue Scale Hypotheses supported 34 correlations > 0.40	On treatment (n = 106) Off treatment < 1 year (n = 41) Off treatment > 1 year (n = 73) P < 0.05 for 3 domains	-
**QOL-CS **[16]	-	Cancer Specific Worry Scale Psychosocial Worry Scale General Health Worry Scale Hypotheses supported 9 correlations > 0.40	Other condition (Y = 28, N = 148) After-effects (Y = 86, N = 90) Income (< $25 K = 36, > $25 K = 127) Gender (F = 101, M = 75) Marital status P < 0.05 for 5 factors	6 factors Represented 56.2% of variance
**PCQL-32 **[60]	Assessed by survivors Review of literature Interviews and pilot test Item wording, relevance, redundancy, reduction [59]	Children' Depression Inventory Stait-Trait Anxiety Inventory-32 (Child) Social Support Scale (Child/Adoles) Self-Perception Profile (Child/Adoles) Child Behaviour Checklist Hypotheses Supported 10 correlations > 0.40 15 correlations < 0.30	On treatment (n = 125) Off treatment (n = 156) P < 0.05 for total scale and 3 domains	-
**PCQL Modular Approach **[62]	Adapted from the PCQL long form and PCQL-32	-	On treatment (n = 125) Off treatment (n = 156) P < 0.05 for the core and symptom modules	-
**PIE **[63]	Assessed by survivors Interviews (n = 15) Item reduction	Rotterdam Symptom Checklist Functional Disability Inventory Restrictions Scale Psychological Symptoms Hypotheses Supported 9 correlations > 0.40 20 correlations < 0.30	Younger children Older Children Maintenance treatment Completed treatment P < 0.05 for 2 domains	-
		SF-36 Functional Evaluation Scale Hypotheses Supported 38 correlations > 0.40 44 correlations < 0.30 [64]		

*Data taken from the publication referenced in the Measure column unless otherwise referenced within the table.

#### Construct/criterion

Five measures examined convergent or divergent validity against other existing measures. Hypotheses were supported by correlations > 0.40 or < 0.30. All of the measures were able to discriminate between known groups. Factor analysis was performed for two measures. None of the measures were examined for criterion (concurrent or predictive) validity.

### Responsiveness

Only two measures reported floor and ceiling effects (Table 7). None of the measures reported their ability to detect clinically important change over time.

**Table 7:** Coding of responsiveness, acceptability and feasibility for each measure.

Measure	Responsiveness	Acceptability	Cross-cultural
**AQoL **[35]	-	Response rate 95% Reading level Flesch-Kincaid grade 6.2 [36]	-
**MMQL Adolescent Forrm **[52]	-	-	Anglicised for UK and shortened to the MMQL-29 [53] Internal consistency in an online format [54] Reliability and validity demonstrated
**PedsQL 3.0 Cancer Module (C&A) **[55]	-	Missing items 0.5%	Initial development in English and Spanish [55] Adapted to Brazilian, German, and Australian cultures [56-58] Reliability and validity demonstrated
**QOL-CS **[16]	-	Response rate 53%	-
**PCQL-32 **[61]	On treatment Floor 1.6-20.0% Ceiling 0%	Response rate 89.5% Missing items 0.01%	-
	Off treatment Floor 1.9-32.7% Ceiling 0%		
**PCQL Modular Approach **[62]	On treatment Floor 0-3.1% Ceiling 3.1-22.9%	Response rate 95% Missing items 0.01%	-
	Off treatment Floor 0-1.9% Ceiling 10.6-35.6%	Reading level Flesch-Kincaid grade 1.8	
**PIE **[63]	-	Reading level Flesch-Kincaid grade 7	-

*Data taken from the publication referenced in the Measure column unless otherwise referenced within the table.

### Acceptability and feasibility

Table 7 also shows that the acceptability of the measures was poorly described with only four measures reporting missing items, and only three measures reporting their reading level. The reading levels that were reported however were appropriate for the population group. Feasibility, the time needed to administer, complete, and score the measure, was not reported for any of the measures.

### Cross-cultural adaptation

Two measures, the MMQL Adolescent Form and PedsQL 3.0 Cancer Module (C&A), have been adapted for cultures other than the United States. For the culturally adapted measures, similar reliability and validity to the original measure was reported. The reliability of MMQL Adolescent Form in an online format has also been verified.

## Discussion

All of the psychosocial measures developed for AYA cancer survivors included in this review showed high total scale internal consistency. However, only one measure reported test-retest reliability coefficients, and although intra-class correlations were reported for the total scale and domains, no item-level test-retest correlations were reported. This may present a problem because while the same overall domain score may be achieved from the first to the second administration, it is possible that the individual item scores that make up the domain score differ between administrations. This may compromise the stability of the measure over time.

Face, content, and construct validity for all of the measures were also psychometrically adequate. However, no measures reported predictive validity. This may reflect difficulties in identifying an appropriate 'gold standard' with which to compare AYA perceptions of their health, or difficulties related to longitudinal study designs such as cost and participant attrition. The implication of this is that the ability of these measures to predict the risk of future health outcomes in AYA cancer survivors remains unknown.

Reporting of measure responsiveness, acceptability and feasibility was poor. No measures reported their ability to detect clinically important change over time, raising questions about the sensitivity of these instruments. Reading level was only reported for three measures. This is of concern because, due to their illness, AYA cancer survivors may have missed a significant proportion of their schooling [15,65]. Poor readability and comprehension of items may lead to misinterpretation, or missing items altogether, thereby reducing the accuracy of results obtained.

Given the absence of findings regarding either test-retest reliability, or responsiveness and acceptability for all of the identified measures, it is difficult to recommend any of them as outcome measures for use in intervention studies. For some, the unknown ability of the measure to remain stable over time would make it difficult to assess whether changes on the measure were due to the intervention alone. For others, the undetermined responsiveness of the instrument would mean that if no change was observed, this could be either due to lack of sensitivity in the measure or lack of an intervention effect.

However, both the MMQL Adolescent Form and the PCQL-32 show promise as measures of quality of life for AYAs. The MMQL Adolescent Form showed good internal consistency (6/7 domains α > 0.70) and test-retest reliability at the domain level (5/7 domains ICC > 0.70). The PCQL-32 also reported good internal consistency, validity and acceptability. Further psychometric testing to establish item-level test-retest reliability and responsiveness for the MMQL, and test-retest reliability for the PCQL-32, is needed.

A literature search did not reveal any other reviews of psychosocial measures for AYA cancer survivors. However, the results of the current review appear to be commensurate with the findings of similar reviews of measures developed for use with other cancer populations. A review of quality of life instruments for use with adult cancer survivors [33]found that, of the nine measures identified, readability, acceptability, feasibility and predictive validity were rarely or (as in the case of predictive validity) never examined. Of the four measures that examined test-retest reliability, only one reported acceptable test-retest coefficients [33]. A comparable review of needs assessment instruments for cancer patients and their families also found that reading levels and sensitivity to change were poorly examined [34]. Similar trends were reported in a systematic review of instruments for the assessment of fatigue in cancer patients [66]. Of 14 instruments identified, only six were examined for test-retest reliability, and only seven analysed responsiveness [66]. In a review of cancer symptom assessment instruments, only one out of 21 identified instruments reported predictive validity [67].

It is interesting to note that all of the multidimensional measures included in this review assessed quality of life in AYA cancer survivors. No measures of perceived need were identified. Using only measures of quality of life may lead to assumptions being made about the type of help AYA cancer survivors would like, rather than allowing individuals to specifically indicate areas in which they would like to receive help [31,32]. In addition, all of the samples used in the development of these measures were recruited through hospitals or medical centres. The extent to which these samples were representative of the broader AYA population, including under-served AYA populations such as those living in rural or remote areas, is unknown.

### Limitations

The literature search for this review was conducted using four online publication databases, and the grey literature was not included. Therefore, it is possible that some relevant measures were missed. However, it is likely that measures identified in this review are likely to be of the best quality as they have been published in peer-reviewed, indexed journals. The step of conducting a second search by measure name would have also minimised the chance that publications relating to relevant measures were overlooked.

The definition of AYA cancer survivors used in this review was young people between the ages of 15 and 30 years. However as a group, the AYA population is not defined well in literature, and ranges from 12 up to 40 years [2-4]. To overcome this discrepancy, any measures developed for an age cohort which overlapped the 15 to 30 year old age bracket were included. This may mean that some of the results reported in this review reflect measure performance with individuals outside the AYA definition used for this review.

## Conclusions

There is a general need to improve the psychometric properties of existing quality of life measures to assess the psychosocial well-being of AYA cancer survivors. The MMQL Adolescent Form and the PCQL-32 have provided the most evidence for their psychometric properties to date. However, without sufficiently robust measures the prevalence of any reported concerns or needs, and the effectiveness of interventions which aim to ameliorate them, remains uncertain. Studies which focus on the test-retest reliability, responsiveness, acceptability, feasibility, and predictive validity of the measure are essential. Development of a psychometrically rigorous measure of perceived needs for use with AYA cancer survivors is warranted.

## Competing interests

The authors declare that they have no competing interests.

## Authors' contributions

AS, KR, and RSF were the initiators of the review. RSF, TCM and MC compiled the psychometric criteria for the review. TCM conducted the database search, coded the abstracts and assessed the psychometric criteria of the measures (reviewer 1). MC cross-checked the inclusion and exclusion criteria for 15% of all identified measures, and confirmed the psychometric assessment for all included measures. All authors contributed to drafting, revising and approving the final manuscript.
